# Tumor Necrosis Factor Inhibitors Exacerbate Whipple’s Disease by Reprogramming Macrophage and Inducing Apoptosis

**DOI:** 10.3389/fimmu.2021.667357

**Published:** 2021-05-20

**Authors:** Asma Boumaza, Soraya Mezouar, Matthieu Bardou, Didier Raoult, Jean-Louis Mège, Benoit Desnues

**Affiliations:** ^1^ Aix Marseille Univ, IRD, APHM, MEPHI, Marseille, France; ^2^ IHU-Méditerranée Infection, Marseille, France

**Keywords:** *Tropheryma whipplei*, Whipple’s disease, macrophages, TNF inhibitor, IFNγ

## Abstract

*Tropheryma whipplei* is the agent of Whipple’s disease, a rare systemic disease characterized by macrophage infiltration of the intestinal mucosa. The disease first manifests as arthralgia and/or arthropathy that usually precede the diagnosis by years, and which may push clinicians to prescribe Tumor necrosis factor inhibitors (TNFI) to treat unexplained arthralgia. However, such therapies have been associated with exacerbation of subclinical undiagnosed Whipple’s disease. The objective of this study was to delineate the biological basis of disease exacerbation. We found that etanercept, adalimumab or certolizumab treatment of monocyte-derived macrophages from healthy subjects significantly increased bacterial replication *in vitro* without affecting uptake. Interestingly, this effect was associated with macrophage repolarization and increased rate of apoptosis. Further analysis revealed that in patients for whom Whipple’s disease diagnosis was made while under TNFI therapy, apoptosis was increased in duodenal tissue specimens as compared with control Whipple’s disease patients who never received TNFI prior diagnosis. In addition, IFN-γ expression was increased in duodenal biopsy specimen and circulating levels of IFN-γ were higher in patients for whom Whipple’s disease diagnosis was made while under TNFI therapy. Taken together, our findings establish that TNFI aggravate/exacerbate latent or subclinical undiagnosed Whipple’s disease by promoting a strong inflammatory response and apoptosis and confirm that patients may be screened for *T. whipplei* prior to introduction of TNFI therapy.

## Introduction

Whipple’s disease (WD) is a rare chronic and systemic disorder, caused by the bacterium *Tropheryma whipplei* and characterized by diarrhea, abdominal pain, and weight loss. Advances in epidemiology and molecular biology have revealed that, beside WD, *T. whipplei* infections cover several clinical entities including localized chronic infections without digestive involvement, acute infections and asymptomatic carriage (1, 2). Although initially thought as a rare disease caused by a rare bacterium, some specific, not yet fully resolved host immune deficiencies explain the rarity of the disease in front of the ubiquity of the bacterium (2, 3).

WD predominantly occurs in white middle-aged men. Histological examination of lesions reveals confluent areas of foamy macrophages strongly colored by periodic acid-Schiff (PAS) staining, containing numerous bacteria and representing the hallmark of the disease (1). However, the first prodromal sign of infection, which typically precedes gastrointestinal signs by several years is arthritis and/or arthralgia (4). As a result, the mean time from joint symptom onset to the diagnosis is 6.7 years (4) and is influenced by immunosuppressive therapy, such as corticosteroids or tumor necrosis factor (TNF) inhibitors (TNFI) (5, 6).

In the last two decades, TNFI have been successfully used to treat and reduce symptoms of both rheumatic conditions such as rheumatoid arthritis, psoriatic arthritis, juvenile arthritis, ankylosing spondylitis and non-rheumatic diseases such as Crohn’s disease, ulcerative colitis, and psoriasis (7). However, the use of such therapies is associated with an increased risk of opportunistic infections and malignancies (8), and also with reactivation of latent tuberculosis or chronic Hepatitis B virus (HBV) infection (9, 10). Several publications have reported the exacerbation of WD or the apparition of gastrointestinal symptoms in patients under immunosuppressive therapy and/or TNFI and for whom the diagnosis of WD was made later (5, 6). In addition, most of the patients who had previous immunosuppressive therapy develop immune reconstitution inflammatory syndrome after effective antibiotic treatment (6). Five TNF antagonists have been developed and approved for clinical use: etanercept, infliximab, adalimumab, certolizumab and golimumab (11). All TNF antagonists are immunoglobulin G1 (IgG1) monoclonal antibodies excepted etanercept, which consists in two extracellular domains of the p75 TNF receptor fused to the Fc portion of a human IgG1. Infliximab is a chimeric mouse/human monoclonal antibody with a murine variable region and human IgG1 constant region, while adalimumab and golimumab are fully human anti-TNF. Finally, certolizumab is an Fab’ fragment of a humanized monoclonal antibody covalently linked to polyethylene glycol (11). All can bind soluble and membrane-bound TNF, but their structural differences account for different mechanistic effects, such as reverse signaling, apoptosis induction, antibody-dependent cell cytotoxicity or complement-dependent-cytotoxicity, both *in vitro* and *in vivo*.

In this study, we aimed at evaluating the effect of etanercept, adalimumab and certolizumab on macrophage responses upon *T. whipplei* infection. We found that all anti-TNF drugs favored *T. whipplei* replication. Surprisingly, TNFI reversed *T. whipplei*-induced M2 macrophage polarization and exacerbate *T. whipplei*-induced macrophage apoptosis. These findings were further confirmed *ex vivo* in intestinal biopsies and in sera from patients that have received or not anti-TNF therapies prior diagnosis of WD. Altogether our results suggest that exacerbation of latent or asymptomatic undiagnosed WD under TNFI is mediated by inflammation and apoptosis and confirm that screening *T. whipplei* infection or carriage should be performed before starting TNFI therapy.

## Materials and Methods

### Cell Culture, Treatment, and Bacteria

Peripheral blood mononuclear cells were isolated by ficoll gradient from buffy coats obtained at the French blood bank after informed consent of the donors according to the convention *n*°7828 established between our laboratory and the “Etablissement Français du Sang” (Marseille, France). Monocytes were then purified by CD14 selection with MACS magnetic microbeads (Miltenyi Biotec) and differentiated into macrophages as previously described (12). Briefly, monocytes were incubated in RPMI 1640 containing 10% human AB serum (Corning) for 7 days. In some experiments, THP-1 cells were cultured in 10% FCS 2 mM glutamine RPMI-1640 and differentiated into macrophages after treatment with 50 ng/ml phorbol-12-myristate 13-acetate (PMA, Sigma-Aldrich) for 48 hours. Macrophages were infected with *T. whipplei* strain Twist-Marseille (CNCM I-2202; bacterium to cell ratio of 50:1) or with 100 ng/ml *Escherichia coli* lipopolysaccharide (LPS, Sigma-Aldrich). The cells were treated with 10 μg/ml etanercept, certolizumab or adalimumab, as previously described (13).

### Patients

Duodenal biopsies from 2 patients were examined retrospectively. Before being diagnosed for WD, both patients were presenting unexplained arthralgias refractory to methotrexate but only one had been further treated with TNFI without improvement. Similarly, serum samples from 12 patients of which 5 had been treated with TNFI before diagnosis were analyzed retrospectively. Patient demographics and characteristics are detailed in **Table 1**. This study was approved by the Local Clinical Ethics Committee of IFR 48 (Marseille, France; n°09-021), all subjects gave their written consent for the use of information and data in the present study.

**Table 1:** Characteristics of Whipple’s disease patients.

	Patient group	
	TNFI	Non TNFI
Number (n)	6	8
Sex (n)	5 males and 1 female	4 males and 4 females
Median age at diagnosis (min - max)	59.5 (39 – 67)	62 (31 – 80)
	Symptoms at diagnosis	
Arthralgia	6	6
Weight loss	3	3
Abdominal pain	1	2
Diarrhea	3	3
Fever	3	1
Adenopathy	3	1
Pericarditis/endocarditis	1	1
Uveitis	0	1
	Treatment before diagnosis	
NSAID	1	0
Corticoids	2	4
Methotrexate	2	4
TNF inhibitors	6	0

### Uptake and Survival of *T. whipplei*

Cells were infected with *T. whipplei* for 4 hours in the presence or not of TNFI, washed to eliminate free bacteria, and incubated for 12 days in 10% FCS 2 mM glutamine RPMI-1640 in the presence or not of TNFI. Bacterial uptake after 4 hours of infection and survival every 3 days for 12 days were assessed by real-time quantitative PCR (qPCR) as previously described (12). Briefly, at the indicated timepoints, cells were lyzed with 1% Triton X-100, and DNA was extracted using the EZNA Tissue DNA Kit (Omega) and qPCR was performed using the SyberGreen Fast Master Mix (Roche Diagnostics) on a CFX96 Touch Real-Time PCR Detection System (Bio-Rad) with primers specific for the *T. whipplei* 16S–23S ribosomal intergenic spacer region (**Table 2**). For each qPCR run, a standard curve was generated using a serial dilution ranging from 10^8^ to 10^2^ copies of *T. whipplei* DNA.

**Table 2:** Primers used in this study.

Target	Forward	Reverse
*ACTB* (human)	ggaaatcgtgcgtgacatta	aggaaggaaggctggaagag
*IL1B* (human)	cagcacctctcaagcagaaaac	gttgggcattggtgtagacaac
*IL6* (human)	ggaaggttcaggttgttttctg	ccaggagaagattccaaagatg
*TNF* (human)	gagggagagaagcaactacagacc	aggagaagaggctgaggaacaag
*CXCL9* (human)	gggagatggtgtggtaattgat	acacttgcggatattctggact
*CXCL10* (human)	tcccatcttccaagggtactaa	ggtagccactgaaagaatttgg
*IL10* (human)	gggggttgaggtatcagaggtaa	gctccaagagaaaggcatctaca
*TGFB1* (human)	tctatgacaagttcaagcaga	gacatcaaaagataaccactc
*IL1RN* (human)	cctaatcactctcctcctcttcc	tctcatcaccagacttgacaca
*CD163* (human)	cggtctctgtgatttgtaaccag	tactatgctttccccatccatc
*IL1R2* (human)	cactcaggtcagggcatactaa	aggagaagaagagacacggatg
ITS (*T. whipplei*)	ccgaggcttatcgcagattg	ggtgacttaacctttttggag

For immunofluorescence, cells were fixed with 4% paraformaldehyde in PBS for 15 min and incubated with rabbit anti-*T. whipplei* antibodies for 45 min followed by anti-rabbit AlexaFluor 555-conjugated antibody for 45 min. AlexaFluor 488-conjugated Phalloidin was used to stain the actin cytoskeleton and 4′, 6-diamidino-2-phenylindole (DAPI, Thermo Fisher Scientific) for nucleus staining. For colocalization studies, the mouse anti-LAMP2 antibody (H4B4, Santa Cruz) was used for 45 min followed by anti-mouse AlexaFluor647-conjugated antibody for 45 min. Coverslips were mounted with Mowiol and observed using an LSM 800 Airyscan confocal microscope (Zeiss) with a 63X oil objective.

### RNA Extraction and qPCR

RNA was extracted using the RNeasy kit (Qiagen), treated with DNase I (Qiagen) before quantification with the NanoDrop Spectrophotometer (NanoDrop Technologies). RNA was retrotranscribed to cDNA using the MMLV-RT kit and oligo(dT) primers (Invitrogen) before qPCR using the SyberGreen Fast Master Mix (Roche Diagnostics) on a CFX96 Touch Real-Time PCR Detection System (Bio-Rad) with the primers listed in **Table 2**. Expression of target genes was estimated based on the endogenous household *ACTB* gene and expressed as fold change (FC) using the following formula: FC = 2^-ΔΔCt^, where ΔΔCt = (Ct_Target_ – Ct*_ACTB_*)_stimulated_ – (Ct_Target_ – Ct*_ACTB_*)_unstimulated_. FC values were then log2-transformed and analyzed by the ClustVis webtool. Expression of target genes was also expressed as relative quantity (RQ) to the endogenous household *ACTB* transcript using the following formula: RQ = 2^-ΔCt^, where ΔCt = (Ct_Target_ – Ct*_ACTB_*).

### Immunoassays

Cell culture supernatants or serum samples were respectively assayed for TNF, IL-1 β, IL-6 and IL-10 (all from R&D Systems) or TNF, IL-10 and IFN-γ (BD Biosciences) by ELISA according to the manufacturer’s instructions.

### Cell Viability and Apoptosis Assays

Cell viability was indirectly estimated by assessing cellular metabolism with 3-[4,5-dimethylthiazol-2-yl]-2,5 diphenyl tetrazolium bromide (MTT) assay. Briefly, cells were stimulated with *T. whipplei* in the presence or not of TNFI for 24 h. Ten μl of MTT (5 mg/ml, Sigma-Aldrich) were added to the cell cultures and incubated at 37°C for 4 hours. The formed formazan crystals were solubilized with 50 μl of dimethylsulphoxide (DMSO) for 30 minutes at 37°C and quantified at 540 nm using a Synergy MxF plate reader (Biotek Instruments). Cell apoptosis was assessed by Annexin-V/7-AAD staining according to the manufacturer instructions (AbCys). Flow cytometry analysis was performed on a BD FACSCanto II (BD Biosciences) and data were analyzed using FlowJo software. Cell apoptosis was also evaluated by immunofluorescence and confocal microscopy after staining with a rabbit anti-active caspase-3 antibody (BD Biosciences) as described above.

### Histology and Immunohistofluorescence

Duodenal tissue was fixed in formalin and embedded in paraffin. Five μm tissue sections were prepared to perform diastase-digested PAS as previously described (14). Apoptosis was evaluated by Terminal transferase deoxytidyl uridine end labeling (TUNEL) using the *In situ* Cell Death Detection Kit, TMR red (Roche) according to the manufacturer’s instructions. For immunostaining, tissue sections were incubated in blocking reagent (3% BSA, 0.5% Triton X-100 in PBS) for 30 min and incubated for 1 h with anti-IFN-γ antibody (BD Biosciences). The images were obtained using an LSM 800 Airyscan confocal microscope (Zeiss) with a 63X oil objective.

### Statistical Analysis

Statistical analysis was performed with the GraphPad Prism Software. Significant differences were evaluated using the Mann-Whitney *U* test and whenever applicable, multiple comparisons were performed by two-way ANOVA followed by the Dunnett’s or Tukey’s posttest or multiple T tests. Differences were considered significant when *P* < 0.05.

## Results

### TNFI Favor *T. whipplei* Replication

**Figure 1:**
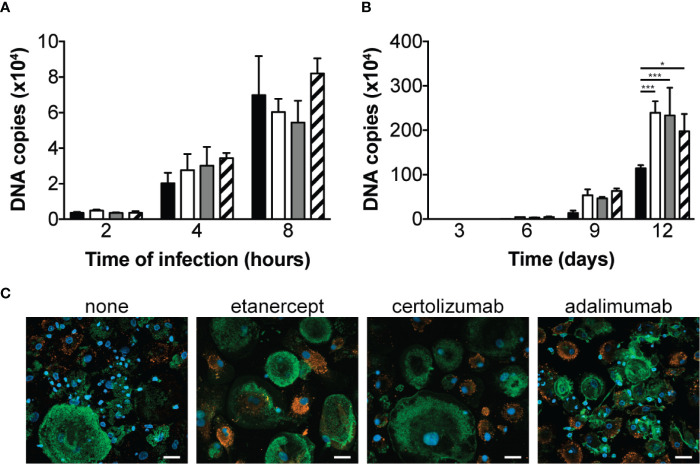
TNFI increase *T. whipplei* replication in macrophages. **(A)** Macrophages were infected with *T. whipplei* (50 bacteria per cell) alone (black bars) or in the presence of etanercept (white bars), certolizumab (grey bars) or adalimumab (hashed bars) and washed after 2, 4 or 8 h. **(B)** Macrophages were infected with *T. whipplei* (50 bacteria per cell) alone (black bars) or in the presence of etanercept (white bars), certolizumab (grey bars) or adalimumab (hashed bars) for 4 h, washed to remove free bacteria and incubated for the indicated periods with etanercept (white bars), certolizumab (grey bars) or adalimumab (hashed bars) or left untreated (black bars). Bacterial DNA copy number was determined by qPCR. **(C)** Representative pictures of infected macrophages incubated for 12 days under the indicated treatment and stained with an anti-*T. whipplei* antibody (red), phalloidin (green) and DAPI (blue), scale bar = 20 μm). The experiment was performed using three different donors (N = 3), and the values represent the mean ± standard error of the mean. * and ***P < 0.05 and 0.001, respectively by two-way ANOVA and the Dunnett’s test for post-hoc comparisons.

We first aimed at determining the effect of different TNF antagonists on macrophage phagocytic and bactericidal activity towards *T. whipplei*. Macrophages were infected in the presence or not of etanercept, certolizumab or adalimumab and bacterial internalization was evaluated by qPCR after 2, 4 and 8 hours. In untreated cells, we observed a time-dependent increase of *T. whipplei* DNA copy number **(** **Figure 1A** **)**. When the cells were treated with etanercept, certolizumab or adalimumab, bacterial uptake was not affected, and similar bacterial loads were measured at every time points **(** **Figure 1A** **)**. Macrophages were next infected with *T. whipplei* for 4 hours in the presence or not of TNFI and bacterial survival was evaluated for 12 days by qPCR. When cells were left untreated, the number of bacterial DNA copy dramatically reduced at day 3 and gradually increased from day 6 to day 12 **(** **Figure 1B** **)**, as previously described (12, 15). As expected, upon etanercept treatment, bacterial replication was significantly increased. Treatment with certolizumab or adalimumab also resulted in significantly increased bacterial DNA copy number, without significant difference between the anti-TNF drug used **(** **Figure 1B** **)**. Analysis of infected cells by confocal microscopy confirmed those results and showed that numerous macrophages in TNFI-treated cultures were heavily infected at day 12 as compared with untreated cells **(** **Figure 1C** **)**, suggesting that increased replication of bacteria did not occur uniformly but rather in a subset of cells. Altogether, these results suggest that TNF blockade favors *T. whipplei* replication in macrophages without affecting bacterial uptake.

### TNFI Repolarize *T. whipplei*-Infected Macrophages

**Figure 2:**
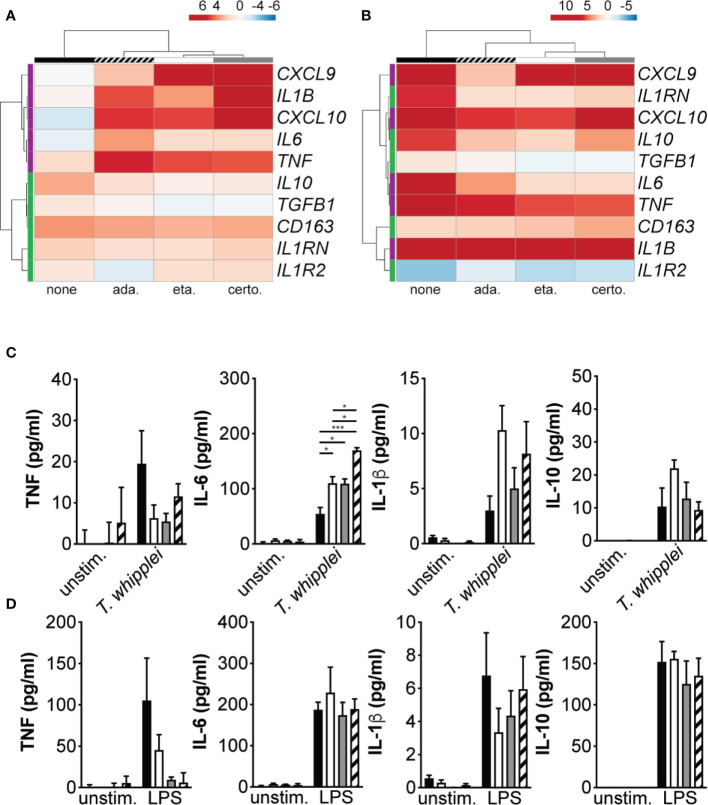
TNFI interfere with macrophage polarization. **(A, B)** Macrophages were stimulated for 6 h with *T. whipplei* **(A)** or LPS **(B)** in the presence or not of etanercept, certolizumab or adalimumab. The expression of macrophage M1 (purple) and M2 (green) polarization genes was investigated by qRT-PCR after normalization to the actin endogenous control and expressed as log2-transformed-foldchanges relative to the appropriate unstimulated condition. The experiment was performed using three (N = 3) or six different donors (N = 6) for *T. whipplei* or LPS stimulation, respectively. The mean log2-transformed-foldchange value was used in the ClustVis webtool to generate the heat-maps. **(C, D)** Macrophages were stimulated for 24 h with *T. whipplei* **(C)** or LPS **(D)** in the presence of etanercept (white bars), certolizumab (grey bars) or adalimumab (hashed bars) or left untreated (black bars) and TNF, IL-6, IL-1β and IL-10 release in the culture supernatants was assessed by ELISA (N = 3). The experiment was performed using three different donors (N = 3), and the values represent the mean ± standard error of the mean. * and ***P < 0.05 and 0.001, respectively by two-way ANOVA and the Tukey’s test for post-hoc comparisons.

In order to understand the mechanisms underlying increased bacterial replication in TNFI-treated macrophages, we measured the expression of M1 and M2 genes following infection. Cells were stimulated for 6 hours with *T. whipplei* or LPS in the presence or not of TNFI and transcript expression was evaluated by qRT-PCR. Hierarchical clustering analysis of transcriptional responses showed that as expected, *T. whipplei* induced the expression of M2 genes, such as *CD163*, *IL10*, *TGFB1*, *IL1RN* and *IL1R2*, while that of the M1 genes investigated (*IL1B*, *TNF*, *CXCL9*, *CXCL10* and *IL6*) remained low or repressed **(** **Figure 2A** **)**. However, when cells were treated with TNF blockers, macrophage responses against *T. whipplei* were markedly different and characterized by a strong induction of M1 genes **(** **Figure 2A**). Some variations were seen depending on the anti-TNF drug used, but the differences seemed rather attributable to the donor rather than the TNFI by itself (**Supplementary FIgure 1** **)**. Interestingly, we did not observe significant differences following TNFI treatment when cells were stimulated with LPS **(** **Figure 2B** and **Supplementary Figure 2**).

We next measured TNF, IL-6, IL-1β and IL-10 in the supernatant from cells stimulated with *T. whipplei* or LPS and treated or not with TNFI. In unstimulated cells, addition of etanercept, certolizumab or adalimumab did not significantly affect basal TNF, IL-6, IL-1β or IL-10 levels **(** **Figure 2C** **)**. When cells were stimulated with *T. whipplei* in the presence of TNFI, TNF secretion was decreased. However, all three anti-TNF drugs significantly increased IL-6 release from macrophages, while that of IL-10 was not significantly modulated. Release of IL-1β was also increased in the presence of TNFI, although not significantly **(** **Figure 2C** **)**. Stimulation with LPS induced the secretion of TNF, IL-6, IL-1β and IL-10 but only TNF release was affected by the presence of TNFI **(** **Figure 2D** **)**. Overall, these data suggest that etanercept, adalimumab and certolizumab interfere with *T. whipplei*-mediated M2 macrophage polarization and cytokine release in response to *T. whipplei*.

### TNFI Favor Macrophage Apoptosis

**Figure 3:**
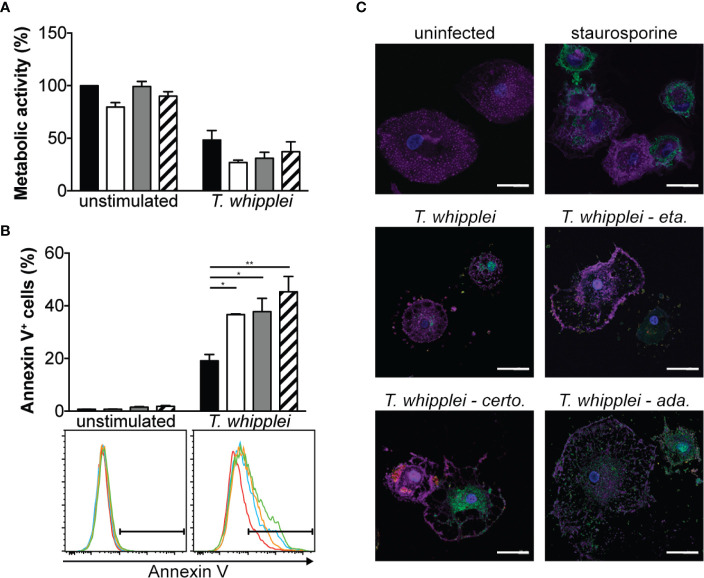
TNFI increase T. whipplei-induced macrophage apoptosis. **(A)** Macrophages were infected or not with *T. whipplei* (50 bacteria per cell) alone (black bars) or in the presence of etanercept (white bars), certolizumab (grey bars) or adalimumab (hashed bars) for 24 h. Cell metabolic activity was assessed by MTT assay and expressed as % of uninfected and untreated cells (N = 3). **(B)** THP-1 macrophages were infected or not with *T. whipplei* (50 bacteria per cell) alone (black bars) or in the presence of etanercept (white bars), certolizumab (grey bars) or adalimumab (hashed bars) for 24 h. Apoptosis was assessed by flow cytometry after annexin V staining (N = 3). Representative plots are shown (red line: untreated; blue line: etanercept; orange line: certolizumab and green line: adalimumab). **(C)** Macrophages were infected or not with *T. whipplei* (50 bacteria per cell) alone or in the presence of etanercept (eta.), certolizumab (certo.) or adalimumab (ada.) for 24 h. As a positive control, cells were treated with staurosporine for 4 h. Representative pictures of macrophages stained with anti-active caspase-3 antibody (green), phalloidin (purple) and DAPI (blue) are shown (scale bar = 20 μm). Values represent mean ± standard error of the mean. * and **P < 0.05 and 0.01, respectively by two-way ANOVA and the Tukey’s test for post-hoc comparisons.

As anti-TNF drugs have been associated with cell apoptosis (16), we next investigated whether etanercept, adalimumab or certolizumab modulate *T. whipplei*-induced apoptosis. We first assessed cell viability by MTT assay which is rather a measure of cellular metabolism. We found that addition of TNFI for 24 h did not significantly alter cell metabolism of uninfected cells **(** **Figure 3A** **)**. Infection of cells with *T. whipplei* induced a significant diminution of cellular metabolism, which may be due to increased cell death. This effect was not significantly affected by the presence of TNFI **(** **Figure 3A** **)**. Second, cell apoptosis was determined using annexin V staining on PMA-differentiated THP-1 macrophages. Addition of TNFI had no effect on uninfected cells after 24 hours **(** **Figure 3B** **)**, confirming data obtained from the MTT experiments. As expected (15, 17, 18), *T. whipplei* induced macrophage apoptosis, but interestingly, treatment of cells with TNFI significantly increased *T. whipplei*-mediated apoptosis **(** **Figure 3B** **)**. These results were further confirmed by investigating activation of caspase 3 in macrophages after incubation with etanercept, certolizumab or adalimumab. As shown in **Figure 3C**, *T. whipplei* alone induced caspase 3 activation at 24 hours. Cotreatment of cells with *T. whipplei* and TNFI increased active caspase 3 staining with a maximal effect obtained when the cells were treated with certolizumab or adalimumab **(** **Figure 3C** **)**. Overall, these results show that TNFI exacerbate *T. whipplei*-induced macrophage apoptosis and that this effect correlates with activation of caspase 3.

### Apoptosis and IFN-γ Are Increased in Patients Diagnosed Under Anti-TNF Therapy

**Figure 4:**
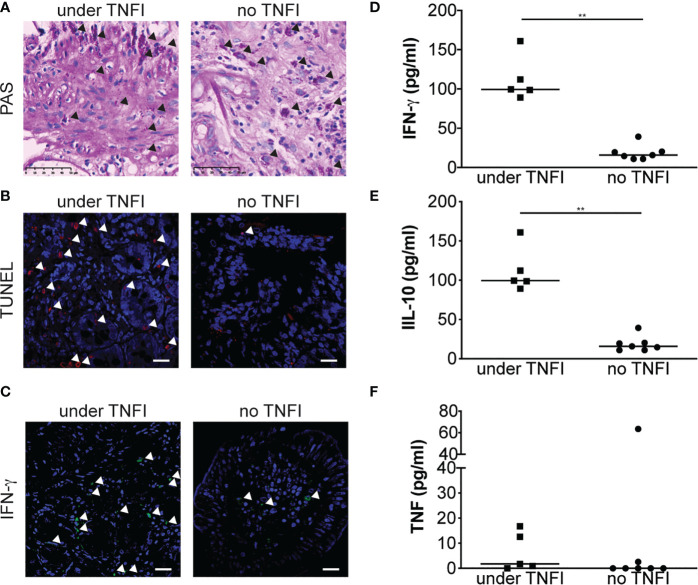
TNFI increase apoptosis in duodenal tissue and local and systemic IFN-γ expression. **(A)** Duodenal biopsy specimen from patients diagnosed under TNFI treatment (left) or not (right) were stained with PAS. Arrows indicate PAS-positive cells (original magnification × 250). **(B)** Cell apoptosis on duodenal biopsy specimen was assessed by TUNEL assay and observed by confocal microscopy. Representative images are shown. Nuclei are visualized in blue after DAPI staining and TUNEL-positive cells (arrows) appear in red, scale bar = 20 μm. **(C)** IFN-γ expression was evaluated by immunohistofluorescence staining and observed by confocal microscopy. Representative images are shown. Nuclei are visualized in blue after DAPI staining and IFN-γ -positive cells (arrows) appear in green, scale bar = 20 μm. IFN-γ **(D)**, IL-10 **(E)** and TNF **(F)** were measured by ELISA in the sera from patients diagnosed under TNFI treatment (square) or not (circle). **P < 0.01 by Mann-Whitney *U* test.

We next extended our results in patients by comparing duodenal biopsy specimens from a patient who was diagnosed for WD while under TNFI and from a patient who was diagnosed for WD but never received TNFI, as control. Immunohistochemical analysis of PAS-stained samples did not reveal obvious differences between the two samples **(** **Figure 4A** **)**. However, when cell apoptosis was assessed, the patient diagnosed for WD while treated with TNFI showed increased number of TUNEL-positive cells in the lamina propria as compared with the control patient **(** **Figure 4B** **)**. We next hypothesized that, as TNFI treatment is associated with M1 macrophage polarization upon *T. whipplei* infection *in vitro*, immune response would be shifted towards a Th1 response in tissues from patient diagnosed for WD during TNFI therapy. Staining for the prototypal Th1 cytokine IFN-γ confirmed our hypothesis and was increased in the duodenal specimen from the patient who was under TNFI therapy as compared with the control patient **(** **Figure 4C** **)**. These results were further extended by analyzing IFN-γ, IL-10 and TNF levels in the sera from patients who had been given (N = 5) or not (N = 7) TNFI before WD diagnosis. In accordance with the results obtained on duodenal tissue, we found that the amount of IFN-γ was significantly higher in the sera from patients who received TNFI therapy before WD diagnosis as compared with control patients **(** **Figure 4D** **)**. Similarly, we found that IL-10 levels were also significantly increased in the sera from patients who received TNFI therapy before WD diagnosis **(** **Figure 4E** **)** while TNF levels were not significantly affected **(** **Figure 4F** **)**. Altogether, these results indicate that the use of TNFI in the setting of WD is associated with increased local cell apoptosis and IFN-γ expression, and increased systemic IL-10 and IFN-γ levels.

## Discussion

Several studies have reported that in the context of undiagnosed, subclinical WD, immunosuppression with corticoids or methotrexate and/or biological therapies such as anti-TNF drugs that are commonly given to treat unclear joint manifestations could accelerate and aggravate the onset of symptoms (5). In a systemic review from 19 publications, 41 patients have been diagnosed for WD while treated for unclear arthropathy with etanercept, infliximab, adalimumab alone or in combination (19). The use of TNF antagonists has been associated with an increased incidence of opportunistic infections, including *Staphylococcus aureus* infections, listeriosis, *Legionella pneumophila* infections, nocardiosis and non-tuberculous mycobacterial infections, cryptococcosis, histoplasmosis, coccidioidomycosis, pneumocystosis, histoplasmosis, candidiasis, and aspergillosis, but also with reactivation of chronic HBV and latent tuberculosis infections (9).

We found that treatment of macrophages with TNFI resulted in increased *T. whipplei* replication. This was associated with the repolarization of macrophages and induction of apoptosis. Increased bacterial replication upon anti-TNF treatment has previously been observed in other infection models. Indeed, TNFI have been shown to promote the intracellular replication of *M. avium* subspecies *paratuberculosis* (20). Similarly, treatment of macrophage with TNF-neutralizing drugs increases the growth of *Legionella pneumophila* (21). *T. whipplei* interferes with phagosome maturation and replicates in a phagosome expressing both early and late markers but lacking acidic hydrolases such as cathepsin D (17). It has been shown that inflammatory cytokines such as IFN-γ or TNF promote phagolysosomal fusion in macrophages (22). Interestingly, in the case of *L. pneumophila*, IFN-γ does not restore anti-TNF-mediated inhibition of rat macrophage microbicidal activities (23). Similar observations were made in human monocytes infected with *L. pneumophila* (24), suggesting that autocrine secretion of TNF potentiates intracellular killing. Therefore, during *T. whipplei* infection, inhibition of TNF may reduce phagolysosome fusion, resulting in increased intracellular replication, as previously shown during *M. tuberculosis* infection (25). However, colocalization studies with the late phagosome marker Lamp2 did not reveal major differences between untreated cells and cells treated with TNFI (**Supplementary Figure 3**). Antimicrobial activity of macrophage towards intracellular bacteria including *M. tuberculosis, L. pneumophila* or *Listeria monocytogenes* is largely mediated by the formation of reactive oxygen and nitrogen species (26–28). As TNF regulates these oxidative mechanisms (29), its inhibition may also favor *T. whipplei* replication through decreased production of reactive oxygen and nitrogen intermediates. Further studies are required to decipher the mechanism by which TNF inhibition supports *T. whipplei* replication.

We previously reported that *T. whipplei* induced macrophage apoptosis and that inhibition of *T. whipplei*-induced macrophage apoptosis was associated with decreased bacterial replication (17, 18). In the present study, we found that macrophage apoptosis was increased when cultures were treated with TNFI, as demonstrated by increased membrane phosphatidyl serine expression and caspase 3 activity. Etanercept and infliximab were previously shown to promote apoptosis of monocytes stimulated by staphylococcal enterotoxin B (30). In addition, infliximab, etanercept, and adalimumab may have cytotoxic effects and induce apoptosis of monocytes and T cells *in vitro* (31). In patients with Crohn’s disease, infliximab induces apoptosis of lamina propria T lymphocytes (32) while in rheumatoid arthritis patients, etanercept and infliximab induce synovial cell type-specific apoptosis in the monocyte/macrophage population, without affecting T cell populations (33). These pro-apoptotic activities may explain at least in part the clinical efficacity of these drugs in inflammatory conditions such as rheumatic arthritis or Crohn’s disease. However, it is tempting to speculate that during *T. whipplei* infections, anti-TNF drugs may worsen the disease and provide a suitable niche for the bacterial replication. Indeed, we previously reported that levels of circulating apoptosis markers were associated with the activity and the severity of the disease (34). In line with these observations, we detected TUNEL-positive cells in the intestinal biopsies from a patient with WD, suggesting that *T. whipplei* induces apoptosis at the systemic level but also locally. Interestingly, apoptosis staining was increased in a patient for whom WD was diagnosed while he was under anti-TNF treatment, confirming our *in vitro* data which evidenced increased macrophage apoptosis in the presence of TNF inhibitors (see above). Similar observations were made in a rabbit model of tuberculosis reactivation, in which etanercept was shown to activate the transcriptional pathways/networks related to cell death, apoptosis and necrosis (35).

We and other have shown that macrophages from WD patients undergo, both in the mucosa and in the blood M2 polarization (36–38). The intestinal anti-inflammatory milieu may exacerbate this M2 polarization, resulting in reduced T cell functions both locally and systemically (36, 38, 39) as revealed by reduced *T. whipplei*-specific Th1 activity (40) and increased activity of regulatory T cells (39). In addition, *T. whipplei* upregulates the expression of Human Leukocyte Antigen-G (HLA-G), which is inversely correlated with that of TNF. Inhibition of HLA-G restores TNF expression while TNF inhibition is associated with increased bacterial replication (12). It was previously described that TNF antagonists induced the formation of immunosuppressive regulatory macrophages producing anti-inflammatory cytokines (41). Other investigators found that TNF blockade with adalimumab inhibited M1 polarization and resumed the M1/M2 ratio both *ex vivo* and *in vitro* in patients with psoriasis (42). These data are in contrast to our current results, in which we found that TNFI revert *T. whipplei*-induced macrophage polarization and promote M1 polarization. These discrepancies might arise from technical differences since in our study, macrophages were simultaneously infected and treated with TNFI while other studies have addressed the effects of TNFI alone (41) or the effect of TNFI on cells that were already polarized (42). Nevertheless, we previously found that TNFI exacerbate M1 polarization in an *in vitro* model of human tuberculous granuloma (13). Similarly, in the rabbit model of tuberculosis reactivation discussed above, etanercept treatment was also associated with a strong pro-inflammatory response (35). Our data are further confirmed by the fact that in the intestinal biopsies from a patient who was diagnosed for WD while under anti-TNF treatment, the staining for IFN-γ (the hallmark of Th1 polarization) was increased as compared with samples from a patient who was diagnosed for WD but never received TNFI. One limitation of our study is the fact that the biopsy specimens were analyzed for only two patients who received or not TNFI. Indeed, *T. whipplei* infections and WD are very rare conditions and thus the availability of such material is limited. However, this increase of IFN-γ was also observed at the systemic level, as revealed by the higher levels of IFN-γ in the sera from additional patients who received TNFI before diagnosis. We also found higher IL-10 levels in the sera from patients who received TNFI before diagnosis, the increase of which may be the reflect of a feedback regulation of inflammation. Hence, inflammation and Th1 polarization in the context of WD may arise from a defect of clearance of apoptotic cells that are increased with anti-TNF. Although phagocytosis of apoptotic cells is usually associated with anti-inflammatory mediators (43), the local increase of danger signals may lead to a prolonged inflammatory response (44). Alternatively, TNFI may restore Th1 responses and IFN-γ expression by counteracting the side effects of chronic TNF exposure as described for rheumatoid arthritis (45), ankylosing spondylitis (46) or moderate psoriasis (47). This anti-inflammatory role of TNF has also been described in murine models of infection including *Corynebacterium parvum* (48) or *M. bovis* BCG (49). Interestingly, TNF blockade in mice chronically infected with *M. tuberculosis* results in a marked pro-inflammatory response involving IFN-γ in the lungs (50).

Taken together, these data showed that TNF blockers favor *T. whipplei* replication in macrophages. This was associated with increased apoptosis and increased inflammation both *in vitro* and *ex vivo*. Interestingly, we did not observed differences between the different TNFI used in our study, while in the case of tuberculosis reactivation, the risk is lower for etanercept than other agents (51). However, this is not the case for WD since no association was made between the class of TNFI and the number of patients later diagnosed for WD (6). Overall, our study highlights the changes induced by TNFI in the context of *T. whipplei* infection. Such changes may constitute or at least contribute to the biological basis of the exacerbation of WD and suggest that in addition to *M. tuberculosis* and HBV, patients with unexplained arthropathy should be screened for *T. whipplei* infection prior to introduction of anti-TNF therapy.

## Data Availability Statement

The raw data supporting the conclusions of this article will be made available by the authors, without undue reservation.

## Ethics Statement

The studies involving human participants were reviewed and approved by Local Clinical Ethics Committee of IFR 48 (Marseille, France; n°09-021). The patients/participants provided their written informed consent to participate in this study.

## Author Contributions

AB acquired, analyzed the data and drafted the manuscript. SM acquired and analyzed the data. DR funded and supervised the study. J-LM provided critical revision of the manuscript, and BD designed and supervised the study, analyzed, interpreted the data and wrote the manuscript. All authors contributed to the article and approved the submitted version.

## Funding

This study was supported by IHU Méditerranée Infection, Marseille, France and by the French Government under the «Investissements d’avenir» (Investments for the Future) program managed by the Agence Nationale de la Recherche (ANR, fr: National Agency for Research), (reference: Méditerranée Infection 10-IAHU- 03). This work was supported by Région Provence Alpes Côte d’Azur and European funding FEDER PRIMI.

## Conflict of Interest

The authors declare that the research was conducted in the absence of any commercial or financial relationships that could be construed as a potential conflict of interest.
