# Supplementary material for: Tumor Necrosis Factor Inhibitors Exacerbate Whipple’s Disease by Reprogramming Macrophage and Inducing Apoptosis
Source: Front Immunol. 2021 May 20;12:667357. doi: 10.3389/fimmu.2021.667357 (PMC8173622; doi:10.3389/fimmu.2021.667357)
Supplement: Supplementary file 2 [file Image_2.pdf]

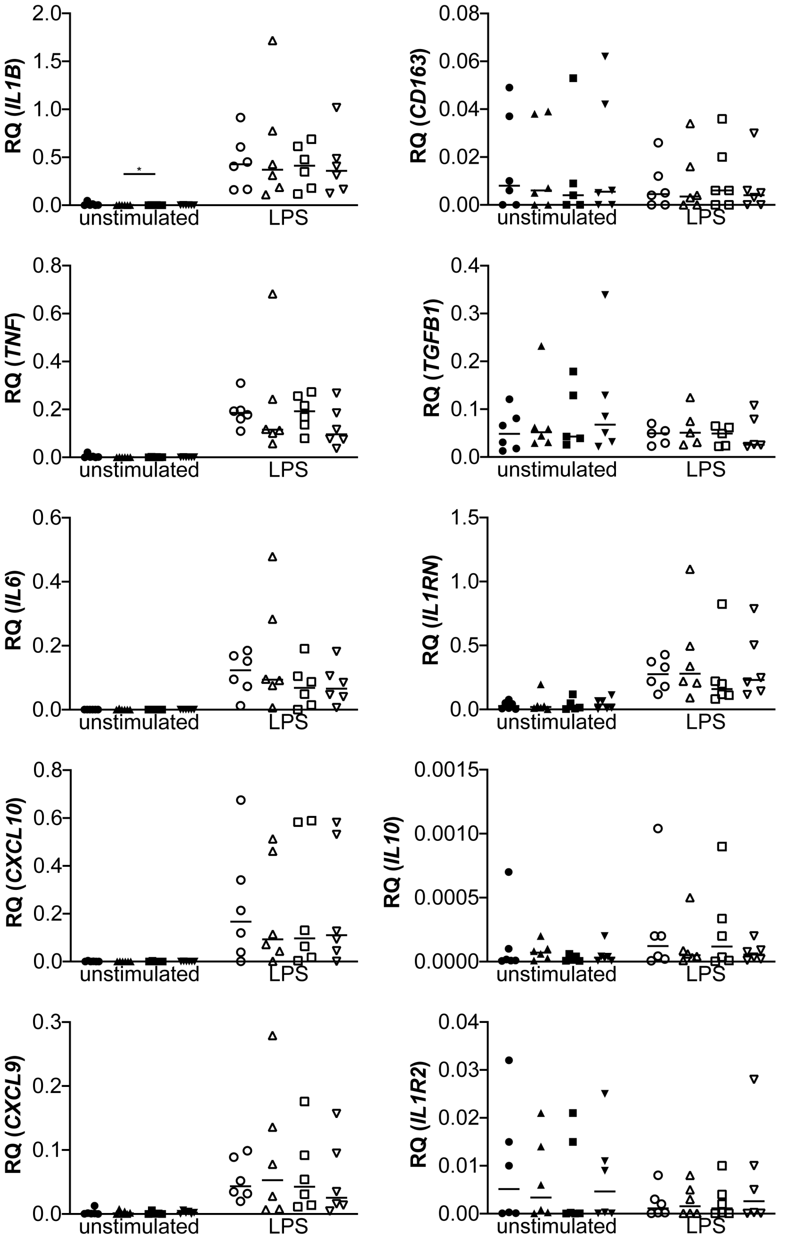

Supplementary figure 2. Macrophages responses to LPS in the presence or not of TNFI. Macrophages were stimulated for 6 h with LPS (open symbols) or left uninfected (black symbols) in the presence or not (circle) of etanercept (up triangle), certolizumab (square) or adalimumab (down triangle). The expression of macrophage M1 (*IL1B*, *TNF*, *IL6*; *CXCL9* and *CXCL10*) and M2 (*CD163*, *TGFB1*, *IL1RN*, *IL10* and *IL1R2*) polarization genes was investigated by qRT-PCR and expressed as relative quantity (RQ) to the endogenous household *ACTB*. The experiment was performed using six different donors (N = 6), bar represents the median. *, $P < 0.05$, respectively by multiple T tests.
