# Supplementary material for: Tumor Necrosis Factor Inhibitors Exacerbate Whipple’s Disease by Reprogramming Macrophage and Inducing Apoptosis
Source: Front Immunol. 2021 May 20;12:667357. doi: 10.3389/fimmu.2021.667357 (PMC8173622; doi:10.3389/fimmu.2021.667357)
Supplement: Supplementary file 3 [file Image_3.pdf]

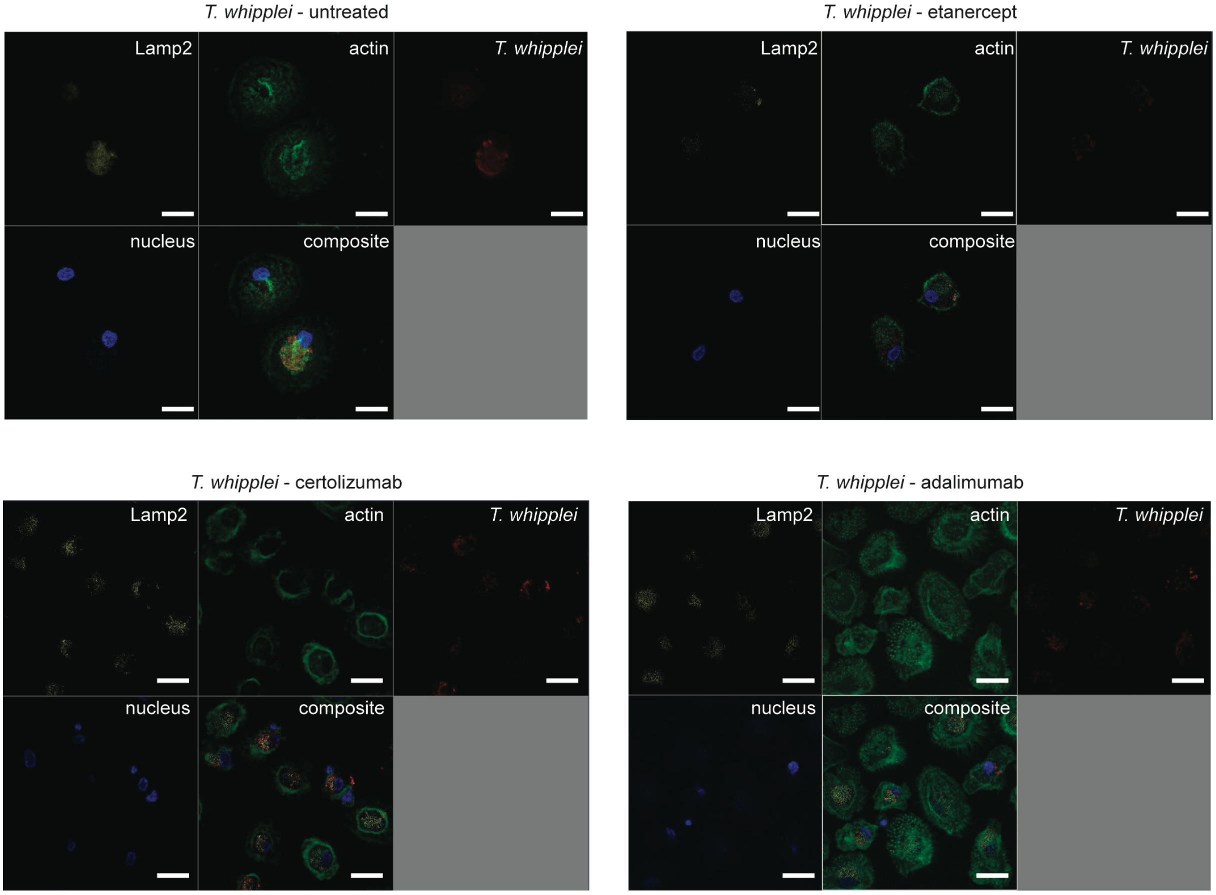

Supplementary figure 3. *T. whipplei*-containing vacuoles in the presence or not of TNFI. Macrophages were infected for 4 h with *T. whipplei* in the presence or not of etanercept, certolizumab or adalimumab and incubated for 24 hours. Representative pictures of cells stained with anti-Lamp2 (yellow), phalloidin (green), *T. whipplei* (red) and DAPI (blue) are shown (scale bar = 20 µm).
